# ATP Release Channels

**DOI:** 10.3390/ijms19030808

**Published:** 2018-03-11

**Authors:** Akiyuki Taruno

**Affiliations:** Department of Molecular Cell Physiology, Graduate School of Medical Science, Kyoto Prefectural University of Medicine, Kyoto 602-8566, Japan; taruno@koto.kpu-m.ac.jp; Tel.: +81-75-251-5311

**Keywords:** ATP, purinergic signaling, ion channel, connexin, pannexin, CALHM, VRAC, VSOR, maxi-anion channel

## Abstract

Adenosine triphosphate (ATP) has been well established as an important extracellular ligand of autocrine signaling, intercellular communication, and neurotransmission with numerous physiological and pathophysiological roles. In addition to the classical exocytosis, non-vesicular mechanisms of cellular ATP release have been demonstrated in many cell types. Although large and negatively charged ATP molecules cannot diffuse across the lipid bilayer of the plasma membrane, conductive ATP release from the cytosol into the extracellular space is possible through ATP-permeable channels. Such channels must possess two minimum qualifications for ATP permeation: anion permeability and a large ion-conducting pore. Currently, five groups of channels are acknowledged as ATP-release channels: connexin hemichannels, pannexin 1, calcium homeostasis modulator 1 (CALHM1), volume-regulated anion channels (VRACs, also known as volume-sensitive outwardly rectifying (VSOR) anion channels), and maxi-anion channels (MACs). Recently, major breakthroughs have been made in the field by molecular identification of CALHM1 as the action potential-dependent ATP-release channel in taste bud cells, LRRC8s as components of VRACs, and SLCO2A1 as a core subunit of MACs. Here, the function and physiological roles of these five groups of ATP-release channels are summarized, along with a discussion on the future implications of understanding these channels.

## 1. Introduction

Adenosine triphosphate (ATP) is abundantly present in the cytosol and used to power energy-consuming reactions, as the hydrolysis of ATP releases energy. Thus, it is known as the energy currency of cells. In addition to this classical cytosolic role, ATP has also been established as an important extracellular ligand of autocrine signaling, intercellular communication, and neurotransmission in numerous physiological and pathophysiological phenomena as it satisfies the criteria for extracellular ligands: production, release, receptors, and extracellular scavenging systems [1,2,3]. ATP is constantly produced within cells through cellular respiration and glycolysis. There are two groups of P2 purinergic receptors, ionotropic P2X and metabotropic P2Y receptors, which respond to ATP [4]. Ectonucleotidases are ubiquitously expressed in the plasma membranes and their active catalytic sites are exposed to the extracellular space to convert extracellular ATP to ADP, AMP, and adenosine [4,5]. Adenosine is then transported back into the cells by nucleoside transporters. Adenosine may stimulate P1 purinergic (A_1_, A_2A_, A_2B_, and A_3_) receptors, thereby exerting as a ligand of autocrine and/or paracrine signaling.

**Figure 1:**
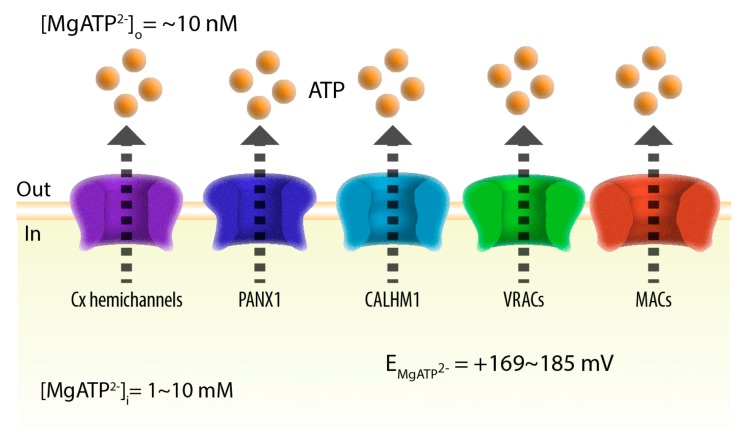
Adenosine triphosphate (ATP) release ion channels. In the presence of physiological levels of Mg^2+^, the majority of ATP molecules exist as MgATP^2−^ anions in both the extracellular and intracellular compartments. Based on the typical extracellular and intracellular MgATP^2−^ concentrations ((MgATP^2−^)_o_ and (MgATP^2−^)_i_, respectively), the equilibrium potential of MgATP^2−^ (E_MgATP_^2−^) was calculated. Cx, connexin; PANX1, pannexin 1; CALHM1, calcium homeostasis modulator 1; VRAC, volume-regulated anion channel; MAC, maxi-anion channel.

Although large and negatively charged ATP molecules cannot simply diffuse across the lipid bilayer of the intact plasma membrane, there are several pathways for both unregulated and regulated ATP release across the plasma membrane. First, cell damage leads to the unregulated leakage of ATP as well as other large cytosolic molecules including enzymes through the disrupted plasma membrane [6,7]. Second, stimulated exocytotic release of ATP occurs in various cell types including neurons and secretory cells. For example, the ATP concentration in neuronal synaptic vesicles is estimated to be up to 100 mM and fast synaptic purinergic neurotransmision occurs in both peripheral [8,9] and central [10,11,12,13] neurons [1,2,3,14]. Importantly, spontaneous and evoked quantal release of ATP has been observed in mouse pyramidal neurons [15]. Physiological relevance of synaptic purinergic neurotransmission in the central nervous system is, however, less clear than in the peripheral nervous system [16]. Third, numerous studies have indicated that ATP can be released through conductive pathways. Considering the steep concentration gradient of ATP anions across the plasma membrane (nano- and millimolar concentrations in the extracellular space and cytosol, respectively) [17,18], the electrochemical potential gradient of ATP should always be outwardly directed at physiological membrane voltages (Figure 1). Indeed, certain channels have been demonstrated to play carrier roles in regulated non-exocytotic conductive ATP release. ATP-binding cassette (ABC) transporter proteins such as cystic fibrosis transmembrane conductance regulator (CFTR) and P-glycoprotein, also known as multidrug resistance protein 1, were previously proposed to function as conductive ATP-releasing pathways [19,20,21]. Although the pore diameter of the CFTR channel can become larger than the size of ATP [22,23], the existence of ABC transporter-mediated conductive ATP transport has been refuted by subsequent studies [24,25]. Currently, five groups of channels are recognized as ATP-permeable channels that mediate various forms of physiological and pathophysiological ATP release: connexin hemichannels, pannexin 1 (PANX1), calcium homeostasis modulator 1 (CALHM1), volume-regulated anion channels (VRACs, also known as volume-sensitive outwardly rectifying (VSOR) anion channels), and maxi-anion channels (MACs) (Figure 1). Recently, several breakthroughs were made in this field. CALHM1 has been identified as a novel ATP-permeable channel that mediates the action potential-dependent release of ATP from taste cells to the afferent gustatory nerves [26,27]. CALHM1 is the first example of voltage-gated ATP channels that can even mediate action potential-dependent fast purinergic neurotransmission. VRACs and MACs had solely been functionally identified by their electrophysiological phenotypes until LRRC8s and SLCO2A1 were recently discovered as their respective core subunits [28,29,30]. Thus, the field of conductive ATP release is entering an exciting era as the identified physiological/physiological roles of ATP-release channels are expanding and their molecular identities are now clearer than ever. This review summarizes the current knowledge on the structures, biophysical properties, and function of the five groups of molecularly identified ATP-release channels.

Another channel, the P2X7 receptor which is a trimeric ATP-gated cation channel, is known to progressively generate a large membrane pore that allow passage of molecules <900 Da following exposure to ATP [31]. There is accumulating evidence of P2X7-dependent ATP release [32,33,34,35]. PANX1 that associates with P2X7 has been proposed to form the large pore [36,37], but normal P2X7 activation-dependent uptake of 375 Da dye observed in PANX1 KO macrophages [38] has challenged the involvement of PANX1, suggesting that P2X7 itself or an unknown protein constitutes the dye uptake pathway. Intriguingly, a very recent study [39] demonstrated that a truncated form of P2X7 lacking both of its amino and carboxyl termini, when reconstituted in proteoliposomes, is sufficient to form a dye-permeable pore. Thus, although it is not discussed in this review, P2X7 is a promising candidate in the family of ATP-release channels.

## 2. Connexin Hemichannels

Connexins are the subunits of vertebrate gap junction channels. More than 20 connexins have been identified in humans and they are widely distributed in various tissues [40,41]. Each connexin protein is named after its predicted molecular weight. For example, connexin 43 (Cx43) is a 43-kDa protein composed of 382 amino acids. Connexin monomers homo- or hetero-hexamerize to form a connexon, also referred to as a hemichannel, which is transported to the plasma membrane. Two hemichannels from two adjacent cells come together and dock end-to-end to form a homo- or heterotypic gap junction that functions as an intercellular pathway connecting the cytoplasms of the two cells both electrically and chemically, and thus it is known as a cell-cell or intercellular channel [40,42,43]. One of the many important biological functions of gap junctions is cell-cell electrical coupling that allows the transfer of action potentials from one cell to a neighboring cell, as seen in cardiac muscles [44]. Human diseases caused by mutations in connexins include X-linked Charcot-Marie-Tooth disease [45], skin diseases [46], non-syndromic deafness [47], and developmental abnormalities [48,49,50], revealing their broad significance. Beyond their roles in gap junctions, the extra-junctional roles of connexins as undocked hemichannels expressed in unapposed plasma membranes are now widely recognized in a variety of tissues [40,51,52]. Most, if not all, connexin hemichannels are activated by positive membrane potentials and their gating is robustly suppressed at physiological concentrations of extracellular Ca^2+^ [53,54,55], and phosphorylation and the redox state as well as a reduction in the extracellular Ca^2+^ concentration ([Ca^2+^]_o_) are known as hemichannel function modulators in physiolgical/pathological contexts [56,57]. Although each connexin subunit confers different permeability properties to the pore of a resulting hemichannel, as demonstrated by different single-channel conductances (15–350 pS [56]), charge selectivities (anion, cation, or no selectivity), and tracer permeabilities, the functionally characterized pores of connexin channels are generally wide (~1.2 nm) enough to allow the passage of a variety of small, soluble, second-messenger molecules, including amino acids and nucleotides [58]. The crystal structure of the human Cx26 gap junction channel [59] revealed that each Cx26 monomer has four transmembrane-spanning domains with cytosolic amino- and carboxyl-termini and homo-hexamerizes to form a Cx26 hemichannel. The amino-terminal helixes of the six subunits line the intracellular pore entrance to form a funnel, the narrowest region of the pore with a diameter of 1.4 nm. Thus, it is suggested that the size and electrical characteristics of the side chains in this funnel region have a marked influence on both the molecular size restriction and charge selectivity of connexin hemichannels. The roles of connexin hemichannels are mainly associated with the release of small signaling molecules into the extracellular space through their pores, and ATP is one of the important permeants of hemichannels [60].

### 2.1. ATP Permeation through Connexin Hemichannels

The first evidence for the involvement of connexin hemichannels in cellular ATP release was derived from stably Cx43- and Cx32-transfected clones of C6 glioma cells that originally lack gap junctions [61]. The Cx43^+^ and Cx32^+^ C6 clones released ~10-fold more ATP compared with wild-type and mock-transfected C6 cells in response to the stimulation of purinergic receptors by UTP. The potentiation of ATP release associated with connexin expression was also noted in other cells including Cx43-, Cx32-, Cx26-, and mutated Cx30-overexpressing HeLa cells and Cx32-overexpressing U373-MG human glioblastoma cells [61]. Lowering [Ca^2+^]_o_ simultaneously triggered the release of ATP and uptake of fluorescent dye tracers (<1 kDa) in Cx43^+^ C6 cells and cells endogenously expressing connexins, such as human and rat astrocytes, human bronchial epithelial cells, and human umbilical vein endothelial cells [62]. Similarly, the heterologous expression of Cx46 or Cx50 enhanced ATP release in *Xenopus* oocytes [63,64]. However, the primary evidence linking connexins with ATP release had been ATP release and tracer uptake associated with connexin expression and their pharmacological sensitivities, and there remained a possibility that connexins are not the conduit for ATP but their expression modulated other ATP-release pathways. ATP permeability of connexin hemichannels was directly demonstrated in Cx43 hemichannels [65]. ATP influx through an excised inside-out membrane patch detected by the luciferase/luciferin-based luminescence assay coincided with single-channel openings of Cx43 hemichannels recorded by patch-clamp recordings. Furthermore, under the inside-out patch-clamp configuration with 130 mM Na_2_ATP in the pipet solution and 280 mM sucrose in the bath, unitary outward currents carried by the influx of ATP anions were observed at positive potentials in the patches obtained from Cx43^+^ C6 cells. The ratio of *P_Na_* to *P_ATP_* was calculated as 1:2.5. These direct observations of ATP permeation through Cx43 hemichannels, together with their large pores (Cx43 forms the largest known pore among connexins), have established Cx43 as an ATP-permeable channel. Thus, despite the lack of direct evidence, other putative ATP-release hemichannels are also considered to function as conductive pathways for ATP anions based on their functional and structural similarities with Cx43.

### 2.2. Physiological Roles of Connexin Hemichannel-Mediated ATP Release

Accumulating evidence has demonstrated the roles of connexin hemichannels as ATP-release channels in a variety of tissues under both physiological and pathological conditions including intercellular Ca^2+^ signaling (Cx43, Cx26/30) [66,67], retinal development (Cx43) [68], renal epithelial ion transport (Cx30) [69,70], central respiratory chemosensitivity in the medulla oblongata (Cx26) [71], and immune responses during inflammation (Cx43) [72]. In the brain, Cx30 and Cx43 are highly expressed in astrocytes but not neurons. ATP released through astrocyte Cx43 has been proposed as a major gliotransmitter for neuron-glia interactions [73,74]. Although many studies investigated the activity of hemichannels in the presence of pathological conditions including ischemic brain injury [75] and inflammation [76], the uptake of ethidium bromide (314 Da) by astrocytes was observed in wild-type but not Cx43 knockout (KO) hippocampal slices [73] under physiological basal conditions, and it was sensitive to pharmacological Cx43 inhibition. Also, the basal extracellular ATP level in the hippocampus was reduced by Cx43 inhibition, indicating that the basal Cx43 activity in astrocytes contributes to the basal ATP level in this brain region [73]. Furthermore, ATP tonically released from astrocytes through Cx43 was demonstrated to act on P2 receptors on CA1 pyramidal neurons to modulate the excitatory synaptic strength [73]. In the olfactory bulb, astrocyte Cx43 opening as well as ATP release depends on neuronal (mitral cell) activity, possibly through a local decrease in extracellular Ca^2+^, and the ATP from astrocytes, in turn, controls spontaneous neuronal activity by acting on A1 receptors following conversion to adenosine [74].

In the auditory cochlea, Cx26 and Cx30 are expressed in supporting cells of the sensory epithelium and in the lateral wall but not in hair cells [77,78]. Mutations of *GJB2* and *GJB6*, encoding Cx26 and Cx30, respectively, are associated with hearing loss [79]. Roles of connexin-mediated ATP release through the interaction of the nonsensory supporting cells with sensory hair cells have been proposed in the developing and mature cochleae [80]. For example, mechanical stimuli induce ATP release from non-sensory supporting cells through hemichannels, and the released ATP acts on P2 purinergic receptors on outer hair cells to reduce their electromotility, which amplifies basilar membrane vibration and consequently enhances hearing sensitivity [81]. Thus, ATP-releasing hemichannels regulate hearing sensitivity. In the prehearing cochlea, the functional maturation of inner hair cells requires spontaneous spiking activity [82], and ATP released from supporting cells through hemichannels plays a role in modulating the spiking pattern in inner hair cells [83,84]. The genetic deletion of Cx30, accompanied by a marked reduction of Cx26 expression, led to the impaired maturation of inner hair cells [84]. Recently, however, another ATP-release channel, PANX1, was found to be widely expressed in nonsensory cells of the cochlear [85], and play roles in cochlear ATP release and hearing based on conditional PANX1 deletion in the cochlea [86,87] (however, a more recent report challenges the involvement of PANX1 in hearing [88]).

## 3. Pannexin 1 (PANX1)

The pannexin (PANX) gene family was discovered as homologs of invertebrate innexin gap junction channels, which are evolutionarily unrelated to vertebrate gap junction-forming connexins [89]. The family consists of three members: PANX1, PANX2, and PANX3, and none of them demonstrably form gap junctions. Rather, their functions as nonjunctional membrane channels have been well-established [90]. Among them, PANX1 has been drawing more attention than PANX2 and PANX3. PANX1 is expressed in various excitable and non-excitable cells including the brain, various epithelial and endothelial cells, erythrocytes, and lymphocytes, whereas the expression of PANX2 and PANX3 is restricted to the brain [91] and skin/bone [92], respectively. In contrast to debates over the functions of PANX2 and PANX3 as plasma membrane channels [93,94,95], there is ample evidence to support the function of PANX1 as a plasma membrane channel. ATP permeability has been most comprehensively established in PANX1 among ATP-release channels and ATP released through PANX1 mediates extracellular auto- and paracrine purinergic signaling in diverse physiological systems. It has yet to be determined whether PANX2 and PANX3 are permeable to ATP. Therefore, this section focuses on PANX1. PANX1 is an *N*-glycosylated protein that has four transmembrane domains with cytosolic amino- and carboxyl-termini. PANX1 monomers homo-oligomerize to form a functional plasma membrane channel. Chemical cross-linking and single-molecule photobleaching approaches suggested a hexameric subunit stoichiometry of a PANX1 channel [96,97]. It is noteworthy that, despite a lack of evolutionary relationships, PANX1, connexins, CALHM1, and LRRC8 share structural features including a membrane topology with four transmembrane domains and hexameric subunit stoichiometry [98].

### 3.1. ATP Permeation through PANX1

The most direct evidence of ATP permeability of the PANX1 channel was obtained in excised inside-out patch membranes of *Xenopus* oocytes injected with human *PANX1* cRNA [99]. When 10:1 outside-to-inside gradients of K_2_ATP were applied to single PANX1 channels, the reversal potential of recorded unitary currents was more negative (~+25 mV) than the equilibrium potential of K^+^ (~+60 mV), suggesting that ATP partly carried the currents. This same study [99] also demonstrated enhanced ATP release induced by high K^+^ exposure from PANX1-expressing oocytes compared with uninjected oocytes. Numerous subsequent studies observed ATP release and the uptake of large molecules <1 kDa associated with PANX1 activation using pharmacological sensitivities and knockdown (KD) and KO of *Panx1* in a variety of native tissues, cell lines, and heterologous expression systems [100,101,102]. Thus, there is firm evidence that PANX1 is an ATP-permeable channel. However, PANX1 does not always allow the passage of ATP. Depolarization-induced whole-cell PANX1 currents were not accompanied by detectable levels of ATP release [103]. A review of the literature reveals marked diversity in the ion permeability and unitary properties of PANX1 [100]. A recent study [63] proposed a compelling model whereby PANX1 forms two open channel conformations depending on the mode of activation: a large-conductance, non-selective, ATP-permeable conformation and an intermediate-conductance, anion-selective, ATP-impermeable conformation. Many physiological stimuli including extracellular K^+^ (K^+^_o_), intracellular Ca^2+^ (Ca^2+^_i_), low oxygen, and mechanical stress (see below) induce both non-selective non-rectifying unitary currents with a large conductance of ~500 pS and permeability to negatively and positively charged molecules larger than ATP. In contrast, exclusively depolarization-activated PANX1 generates outwardly rectifying anion-selective unitary currents with ~75 pS at positive potentials and ~15 pS at negative potentials [63,103,104]. Remarkably, this intermediate-conductance conformation of the channel does not confer ATP permeability. Different reactivities of the terminal cysteine to thiol reagents and single-particle electron microscopic analysis suggested two distinct channel structures associated with the two different biophysical channel properties. Thus, the high-level conductance may be an essential requirement for the ATP permeability of the channel [63]. However, a more recent study [97] challenged this conclusion. PANX1 activated by truncation of the carboxyl-terminal auto-inhibitory region exhibited outwardly rectifying intermediate conductance currents (~90 pS and ~15 pS at positive and negative potentials, respectively) associated with ATP release and TO-PRO-3 uptake. In summary, the ATP permeability of PANX1 depends on the mode of activation, and its electrophysiological fingerprints remain unclear.

### 3.2. Physiological Roles of PANX1-Mediated ATP Release

PANX1 can be reversibly and irreversibly activated by diverse mechanisms (comprehensively reviewed in [100,101]) to release ATP and play many important physiological and pathophysiological roles by mediating extracellular purinergic signaling. (i) It is currently controversial whether the channel-gating is voltage-dependent. While depolarization-induced increases in the open probability (*P_o_*) were reported in basally-active PANX1 channels [103,104], the *P_o_* of the caspase-activated channels was unaffected by a membrane voltage between −80 and +80 mV [97]. PANX1 does not possess the canonical voltage sensor domain. A recent study [104] that assumed the anion selectivity of the channel proposed an unusual gating mechanism whereby the mean open time of unitary PANX1 currents depends on the direction and amplitude of anion flux through the channel, leading to the apparent voltage-dependent gating. The voltage or current direction/amplitude sensitivity may also be activation mode-dependent. Nevertheless, the membrane voltage may not be a physiological stimulus of PANX1 as an ATP-release channel [63] because the high positive membrane voltages required to activate PANX1 occur only in excitable cells and, more importantly, voltage-activated PANX1 is not permeable to ATP. (ii) PANX1 channels are mechanically activated. The *P_o_* of human PANX1 expressed in *Xenopus* oocytes increased when stressed mechanically by suction applied to cell-attached and excised membrane patches [99]. Mechanically-activated PANX1 contributes to ATP release from airway epithelia induced by air-puff stimulation [105], from erythrocytes induced by hypotonic cell swelling [106], from metastatic breast cancer cells induced by mechanical deformation [107], and from distended urothelial cells [108]. The physiological roles of mechanically-stimulated ATP release from PANX1 include airway defense [105], local blood-flow regulation [106], and micturition [108]. During metastatic progression, most circulating cancer cells die from microvasculature-induced cell deformation at end organs. A recent study [107] identified a channel-activating mutation encoding a truncated form of PANX1, PANX^1–89^, in metastatic breast cancer cells that promoted cancer cell survival following mechanical trauma and thereby metastasis by enhancing the release of ATP, an autocrine suppressor of deformation-induced apoptosis. Thus, PANX1 is a potential target for anti-metastatic drug development. (iii) Ca^2+^_i_ can directly open PANX1, as demonstrated by increased channel activity in response to the application of Ca^2+^ to the cytoplasmic face of the channels in inside-out patch membranes [109]. ATP release following the activation of P2Y receptors [109], AT1 receptors [110], and thrombin receptors [111,112] is attributed to Ca^2+^_i_-activated PANX1 following Ca^2+^ release from the endoplasmic reticulum. However, Ca^2+^_i_ activation of PANX1 was not observed in PANX1-transfected HEK293 cells [113], suggesting that Ca^2+^_i_ activates the channel only under certain conditions. Unlike most connexin hemichannels and CALHM1, PANX1 gating is not affected by extracellular Ca^2+^ [114]. (iv) K^+^_o_ is also known as a strong activator of PANX1. Increased K^+^_o_, which can occur under pathological conditions such as ischemic injury, activates PANX1 channels in a depolarization-independent manner [63,115,116]. Similarly to Ca^2+^_i_ activation, this K^+^_o_ activation was not observed in PANX1-transfected HEK293T cells [100]. (v) Extracellular ATP activates PANX1 through the activation of P2X7 [37] or P2Y receptors [109,117]. Whereas the P2Y-mediated PANX1 activation involves Ca^2+^_i_ [109,117], the P2X7-mediated activation is not dependent on Ca^2+^_i_ [36] but through their close association [37]. Remarkably, extracellular ATP also inhibits PANX1 at concentrations higher than those required for activation [118,119], possibly through direct binding to the putative binding site located in the first and second extracellular loops [120]. Extracellular ATP-induced activation and inhibition of PANX1 are proposed to provide mechanisms underlying ATP-induced ATP release (i.e., positive-feedback amplification) and its negative feedback regulation at lower and higher extracellular ATP concentrations, respectively. (vi) Post-translational modifications also modulate channel activity. Src kinase-mediated tyrosine phosphorylation following the activation of NMDA receptors [121,122,123], TNFα receptors [124], and P2X7 receptors [125] activates PANX1, whereas nitric oxide inhibits the channel through *S*-nitrosylation [126] and PKG-mediated phosphorylation [127]. (vii) Adding to the above that are all reversible, other reversible activation mechanisms include low oxygen stress [106,128] and the activation of other receptors including thromboxane receptors [129], α1 adrenergic receptors [130], and insulin receptors [131]. In adipocytes, insulin receptor activation causes PANX1 opening to release ATP, which supports insulin-induced glucose uptake [131]. (viii) PANX1 is irreversibly activated by the caspase 3/7/11-mediated cleavage of its carboxyl-terminal tail, which acts as an auto-inhibitory region [97,132,133,134]. Apoptotic cells release ATP and UTP as “find-me” signals through caspase 3/7-activated PANX1 channels to recruit phagocytes for the clearance of dying cells [132,135]. Lipopolysaccharide-induced pyroptosis involves the activation of caspase 11 and cleavage activation of PANX1, which leads to ATP release and ultimately cell death [134]. (ix) Interaction of PANX1 with other membrane proteins such as P2X7 [37], K_V_β3 [136], and Ca_V_1.2 [137] has been identified. Interaction with P2X7 and K_V_β3 respectively links the extracellular purinergic signaling to PANX1 activation [37], and changes the pharmacological sensitivities of PANX1 [136], whereas PANX1 changes the pharmacological sensitivities of a splice variant of Ca_V_1.2 to clevidipine [137]. In rats, naloxone-induced withdrawal from morphine was found to activate PANX1 in microglia of the spinal cord, possibly following the activation of P2X7, and induce PANX1-mediated release of ATP, which is a key substrate for the aversive symptoms of opiate withdrawal [138]. Importantly, the clinically used PANX1 blockers, mefloquine and probenecid, suppressed ATP release and the severity of withdrawal without affecting opiate analgesia [138], suggesting PANX1 as a potential therapeutic target for alleviating withdrawal symptoms.

## 4. Calcium Homeostasis Modulator 1 (CALHM1)

In 2008, *CALHM1*, previously termed *FAM26C*, was discovered in a bioinformatics search for human genes preferentially expressed in the hippocampus and located in the susceptible loci of Alzheimer’s disease [139]. *CALHM1* encodes a 346-amino acid *N*-glycosylated plasma membrane protein that regulates plasma membrane Ca^2+^ permeability. A nonsynonymous polymorphism in the *CALHM1* gene, P86L, was found to influence the age at onset of Alzheimer’s disease, possibly by altering amyloid beta peptide levels [139,140]. Subsequent studies established CALHM1 as a pore-forming subunit of a plasma membrane voltage-gated non-selective ion channel with an ion-conducting pore wide enough to accommodate ATP molecules [27,141,142]. Its unitary currents show a linear, non-rectifying current-voltage relationship with single-channel conductance of ~24 pS. At a physiological [Ca^2+^]_o_, CALHM1 is closed at the resting membrane voltage and needs strong depolarization to open (*V*_1/2_ ~82 mV at 5 mM [Ca^2+^]_o_), whereas in the absence of extracellular divalent cations, CALHM1 can be opened at more negative membrane voltages (*V*_1/2_ ~−76 mV), demonstrating that CALHM1 gating is activated by both Ca^2+^_o_ reduction and depolarization [141]. Recently, *S*-palmitoylation, a reversible attachment of palmitate to Cys residues, at two intracellular Cys adjacent to the third and fourth transmembrane domains was demonstrated to modulate both the voltage sensitivity and lipid-raft association of the channel [143]. Other modes of CALHM1 activation remain to be identified. To date, among six members (CALHM1–6) of the CALHM family, only CALHM1 is known to form a functional ion channel. It has been suggested to be involved in a number of physiologically important ATP-release phenomena in taste buds and airway epithelial and urothelial cells.

### 4.1. ATP Permeation through CALHM1

A CALHM1 monomer exhibits the membrane topology of four transmembrane domains, intracellular amino- and carboxyl-termini, one intracellular loop, and two extracellular loops. Single-molecule photobleaching and non-denaturing Blue Native-PAGE experiments suggested that the functional CALHM1 channel is a homohexameric complex of the monomers [142]. The CALHM1 channel pore exhibits weak ion selectivity with *P_Na_/P_K_/P_Ca_/P_Cl_* of human CALHM1 = 1:13.8:1.14:0.52 [141,142,144]. It is notable that both cations and anions permeate through the channel. The weak ion selectivity is thought to be due to its wide permeation pore. By measuring the relative permeabilities of various tetraalkylammonium ions, the functional diameter of the pore of CALHM1 was estimated to be approximately 1.42 nm at its narrowest region [142]. Independent optical analyses of fluorescent dye uptake using dyes of the same valence and similar structure with different sizes confirmed this pore size estimate. Thus, the permeation pore of CALHM1 is large enough to accommodate ATP molecules (1.14–1.22 nm).

Although direct electrophysiological measurements of ATP currents through CALHM1 are lacking, ATP release associated with CALHM1 expression and function has been demonstrated by measurements of ATP release from CALHM1-expressing cells in vitro and in vivo [27,105,145]. Heterologous expression of CALHM1 in HeLa cells, COS cells, and *Xenopus* oocytes led to the cell plasma membranes showing ATP permeability. CALHM1-transfected HeLa cells released ATP into the extracellular milieu in response to stimuli that can activate CALHM1 gating, including reduction of [Ca^2+^]_o_ and membrane depolarization. Inhibitory effects of Ca^2+^_o_ on CALHM1 gating and ATP release are similar with apparent half-maximum inhibitory [Ca^2+^] (IC_50_) of ~220 and 490 µM and Hill coefficients of 2.1 and 1.9, respectively [27,141]. The CALHM1-associated ATP release is sensitive to ruthenium red, a CALHM1 channel blocker, but not to inhibitors of other cellular ATP-release pathways, including connexins, PANX1, and VRAC. Also, ATP release was abolished in type II taste bud cells [27] and nasal epithelial cells [105] in *Calhm1* KO mice. Together with the characteristics of the CALHM1 permeation pore, including the notable anion permeability and size larger than ATP, these observations strongly suggest that ATP can permeate through the pores of activated CALHM1 channels.

### 4.2. Physiological Roles of CALHM1-Mediated ATP Release

The physiological significance of CALHM1 had remained elusive until the discovery of its ATP-releasing function. CALHM1 has been reported to be expressed in the brain [139,141,146], taste buds [27,143,147], nasal epithelium [105], bladder [145], other tissues [139]. In the human and mouse brains, its expression has been detected in hippocampal and cortical neurons, where it has been suggested to be involved in the [Ca^2+^]_o_ regulation of neuronal excitability by modulating membrane conductance [141], and in memory flexibility by modulating long-term synaptic potentiation via the phosphorylation of NMDA and AMPA receptors mediated by Ca^2+^ influx through the channel [148]. It is currently unknown whether CALHM1 plays roles as an ATP-release channel in the brain.

**Figure 2:**
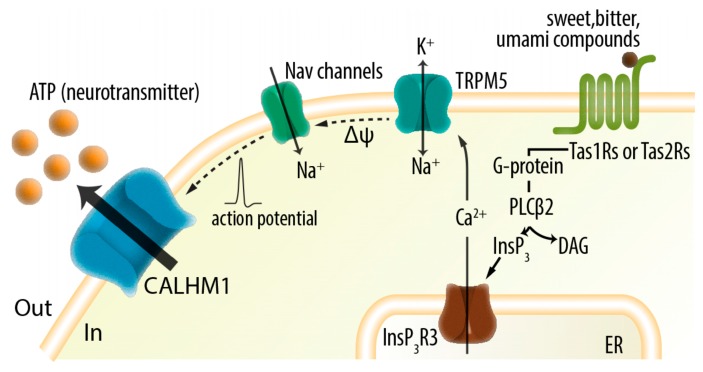
Action potential-dependent ATP release from type II taste bud cells through voltage-gated CALHM1 channels mediates fast purinergic neurotransmission of sweet, bitter, and umami tastes. Δψ, receptor potential; ER, endoplasmic reticulum; DAG, diacylglycerol; InsP_3_, inositol 1,4,5-trisphosphate; PLCβ2, phospholipase β2.

The physiological contribution of CALHM1 as a cellular ATP conduit is best described in the taste buds primarily located on the tongue. Each taste bud typically composed of 50–100 cells contains three cell types: type I, II, and III. Type II cells mediate the sensation of sweet, umami, and bitter (and high salt) tastes. Gustatory stimulation leads to type II cell excitation, i.e., action potential firing. ATP released from taste bud cells has been established as a signaling compound essential for the perception of all taste qualities. Genetic deletion and pharmacological blockade of ATP-gated P2X2/3 receptor cation channels expressed in the afferent gustatory nerves led to mice lacking responses to all taste qualities [149,150]. ATP release in response to taste stimuli has been detected from type II cells, whereas it is still unclear whether the other cell types release ATP [151,152,153]. Thus, ATP currently fulfills the requirements as a neurotransmitter in type II cells: (1) its presence (ATP is the universal currency in all cells); (2) its release; and (3) the presence of specific receptors in the afferent nerve fibers (P2X2/3 receptors). Notably, type I cells that wrap around other cell types have been demonstrated to express ectonucleotidase NTPDase2 and play significant roles in the effective removal of released ATP to maintain appropriate neurotransmission [154]. Because type II cells do not possess conventional synaptic contacts with afferent nerves, they had been suggested to utilize a non-exocytotic, ion channel-mediated mechanism for the release of ATP [151,153,155]. In the primate and murine taste buds, *Calhm1* mRNA was found to be exclusively expressed in type II cells [27,147]. Cre recombinase expression was confined to type II cells in *Calhm1^V5-ires-Cre^* mice that express Cre under the control of the *Calhm1* promoter [143]. Furthermore, CALHM1 currents were detected almost exclusively in type II cells [27]. Collectively, despite a lack of evidence of protein expression these findings suggest that CALHM1 expression is restricted in type II cells in taste buds. In mice, *Calhm1* KO markedly reduced a voltage-gated outward current recorded in isolated type II cells that mediates ATP release [151,156] and abolished gustatory stimuli-evoked tetrodotoxin-sensitive ATP release from taste buds. Consequently, *Calhm1* KO mice specifically lacked responses to sweet, umami, bitter, and high-salt tastes [26,27,157]. These observations established CALHM1 as an essential component of the type II cell ATP-release channel (Figure 2).

However, the ATP release channel complex in type II cells remains unknown. In the presence of physiological concentrations of extracellular Ca^2+^, the activation of heterologously-expressed CALHM1 (τ ~ 3 s at +60 mV) is too slow to be activated by rapid action potentials [141,158], and the native ATP channels activate much faster (τ ~ 10 ms at +60 mV) [141,156,158]. The type II cell ATP release but not the expressed CALHM1 channel is blocked by carbenoxolone [141,152,153,159]. Furthermore, there is also evidence for the Ca^2+^_i_ dependency of taste-evoked ATP release from type II cells [160], but CALHM1 is insensitive to Ca^2+^_i_ [141]. PANX1 was proposed as a prime candidate for the taste cell ATP channel [153] because it is activated by depolarization and Ca^2+^_i_ [109], inhibited by carbenoxolone [114], and insensitive to extracellular Ca^2+^ [114]. However, normal type II cell ATP release and taste perception in PANX1 KO mice [103,161,162] indicate that PANX1 is not necessary. Although connexin hemichannels have also been proposed as the ATP conduit in type II taste cells, their involvement is unclear [151]. Taken together, CALHM1 is a necessary subunit of the native ATP-release channel but it needs to be associated with other proteins and/or undergo modifications to gain the specific biophysical and pharmacological properties of the native channel [26,163]. Thus, whereas CALHM1 by itself can form an ATP-release channel, its regulatory mechanisms likely play indispensable roles to facilitate its physiological functions. *S*-palmitoylation that was detected on CALHM1 proteins in taste cells is suggested to be involved, but this post-translational modification alone cannot fully bridge the gap between CALHM1 function in vitro and in vivo [143]. A compelling hypothesis proposes that CALHM1 and PANX1 associate each other to form an ion channel in salt-sensing taste cells in fungiform taste buds [164]. In rat fungiform salt-sensing taste cells identified by the presence of both an amiloride-sensitive current and a voltage-gated Na^+^ current, a “CALHM1-like” current identified by pharmacological sensitivities and slow activation and deactivation kinetics similar to those of CALHM1 was reported [164], suggesting the presence of CALHM1. This “CALHM1-like” current was augmented by drugs that inhibit PANX1 (carbenoxolone, probenecid, and BzATP), which led to the above hypothesis [164]. However, more studies are clearly required to support this hypothesis. For example, molecular evidence of CALHM1 and PANX1 expression and interaction in salt-sensing fungiform cells is lacking. Despite the actions of inhibitors, PANX1 currents were not detected in those cells. Note that carbenoxolone, probenecid, and BzATP are specific to PANX1 only among connexins, PANX1, and CALHM1, but they all have been shown to affect other ion channels/transporters. It is also unclear whether the “CALHM1-like” channel is permeable to ATP or why the proposed CALHM1-PANX1 interaction only occurs in salt-sensing cells because probenecid does not enhance CALHM1 current in type II cells [27] which express PANX1 [153].

Other possible roles of CALHM1 as an ATP-release channel have recently been reported in the urinary bladder [145] and nasal epithelial cells [105]. In the urinary bladder, the distension of urothelial cells caused by bladder filling leads to the release of ATP, which activates P2X2/3 receptors on the suburothelial sensory nerves and thereby controls pain responses and afferent pathways controlling voiding reflexes [165,166,167,168]. In addition, released ATP also binds to P2 receptors on the umbrella cells to stimulate membrane insertion at the apical membrane of these cells, resulting in an increase in the apical surface area and thereby a reduction in the epithelial membrane tension [169]. However, the mechanisms underlying distension-induced urothelial ATP release are largely unknown. CALHM1 expression was identified in the urothelium as well as the suburothelium and detrusor muscle of the porcine bladder by RT-PCR and immunohistochemistry [145]. Both hypotonic stress and the depletion of Ca^2+^_o_ induced ATP release from cultured urothelial cells that were significantly inhibited by ruthenium red, a nonspecific CALHM1 blocker, and a specific CALHM1 antibody [145], suggesting roles for CALHM1 in urinary bladder purinergic signaling along with other ATP-release mechanisms including exocytosis, connexins, and PANX1 that are also present in urothelial cells [108,170]. However, because the immunogen of the CALHM1 antibody used in this study (HPA018317, SIGMA-ALDRICH) is a region in the intracellular carboxyl terminus of CALHM1, the inhibitory effects of this antibody on urothelial ATP release when added to the extracellular solution are difficult to interpret. As Gd^3+^ can block other ion channels, further studies using *Calhm1* KO animals are required to validate the roles of CALHM1 in urothelial ATP release. In the airway epithelium, effective mucociliary clearance achieved by ciliary beating is a major defense mechanism of the respiratory tract [171,172,173,174]. Although mechanical forces [175,176,177,178,179] are known to stimulate ATP release from the airway epithelium, which leads to an increase in the ciliary beat frequency as autocrine/paracrine signaling [180], the ATP-release mechanisms remain elusive. CALHM1 transcripts were detected in mouse nasal septal epithelial cells grown at an air-liquid interface, and apical ATP release following an air puff or membrane depolarization was significantly reduced in *Calhm1* KO cells. Notably, carbenoxolone, a PANX1 blocker, completely inhibited the remaining air puff-induced ATP release from *Calhm1* but not *Panx1* KO cultures, suggesting that CALHM1 and PANX1 both work to mediate mechanically-stimulated apical ATP release. Intriguingly, these two studies involving the urothelium and airway epithelium suggest the mechanical activation of CALHM1 channel gating, which has not been directly demonstrated. As CALHM channel research is still in its infancy, future studies will likely elucidate more physiological roles of CALHM1 as a cellular ATP-release pathway in other tissues.

## 5. Volume-Regulated Anion Channels (VRACs)

Regulation of the cell volume is of marked importance for most cell types. Hypotonic stress, i.e., a reduction in extracellular osmolarity, causes cellular swelling via the influx of water across the plasma membrane. In response to hypotonic cell swelling, cells activate several mechanisms to restore the normal cell volume, termed regulatory volume decrease [181]. Although various effectors are involved in the regulatory volume decrease depending on cell types, the major mechanism is the conductive exit of organic and inorganic osmolytes through ion channels called VRACs, also known as VSOR anion channels. The functional characteristics, regulation, and roles of VRACs have been extensively studied for decades [182,183,184,185], but the molecular identification of VRACs (LRRC8 heteromers) was only recently accomplished [28,30]. VRAC currents exhibit anion selectivity with the permeability sequence of I^−^ > Br^−^ > Cl^−^ > F^−^, moderate outward rectification, inactivation at positive membrane potentials, and an intermediate unitary conductance with a slope conductance at 0 mV of 30–75 pS [186,187]. Intracellular ATP, probably through direct binding to the channels, is required for their activity [188,189]. VRACs are ubiquitously expressed in many cell types [190]. Hypotonic cell swelling triggers VRAC activation via a reduction in the cytoplasmic ionic strength [191,192], whereas they can also be activated via a reactive oxygen species-mediated mechanism by pro-apoptotic compounds such as cisplatin [193] and by the activation of plasma membrane receptors, including purinergic [194], metabotropic glutamate [195], epidermal growth factor [196] and bradykinin [197] receptors.

Recently, two groups independently identified proteins responsible for VRACs through genome-wide RNAi screening [28,30]. Heteromers of LRRC8A and other LRRC members (LRRC8B, C, D, and E) are crucial VRAC components. KO [30] and KD [28,30] of LRRC8A expression ablated VRAC currents, establishing LRRC8A as an essential component of VRACs. In cells engineered to lack all five LRRC8 members (LRRC8A to LRRC8E), VRAC currents were not restored by LRRC8A transfection alone, whereas cotransfection of LRRC8A and one of the other LRRC8 homologs restored VRAC currents [30], demonstrating that LRRC8A and at least one other LRRC8 homolog are required to form functional VRACs. Importantly, point mutations in LRRC8A cause changes in the anion selectivity of VRAC currents [28], and the reconstitution of LRRC8 complexes in bilayers is sufficient to form anion channels activated by osmolality gradients [192], demonstrating that LRRC8 proteins form the VRAC pore. Three or more different LRRC8 subunits may be contained in individual VRACs [198,199]. The subunit composition of LRRC8 heteromers determines the inactivation kinetics [30], unitary conductance [192], sensitivity to oxidative stress [200], and substrate selectivity [198,199,201,202]. LRRC8 proteins have four membrane-spanning domains with cytosolic amino- and carboxyl-temini [30]. Their carboxyl-termini contain up to 17 leucine-rich repeats. The sequence homology between LRRC8 and PANX1 also suggests that LRRC8 proteins form hetero-hexameric channels [203]. Recently, the single-molecule photobleaching approach was employed to estimate the number of each subunit in the LRRC8A/E heteromer in *Xenopus* oocytes injected with fluorescently-tagged LRRC8A and E [198]. The number of bleaching steps of each subunit showed a broad distribution that fits well with the Poisson but not binominal distribution, indicating variable subunit stoichiometry. Also, the average numbers of LRRC8A and E within heteromers were calculated to be ~3 and ~2.5, respectively, suggesting that the average total number of subunits is more than 5. However, it remains unknown whether the total number of subunits in LRRC8 channels is fixed. Considering the various LRRC8 combinations and variable stoichiometry within a channel complex, LRRC8 proteins can potentially form numerous VRACs with both shared and distinct functions. It should also be noted that LRRC8s are essential but not sufficient components for the generation of VRAC currents, and additional factor(s) remain to be identified [204].

### ATP Permeation through VRACs

VRACs are permeable to organic osmolytes such as taurine and glutamate. Independent measurements of release from voltage-dependent permeating block of VRAC currents by calixarenes of different sizes at high membrane voltages [205,206] and the partitioning of non-electrolytes (tri- and poly-ethylene glycols) into the pore of VRACs [207] led to an estimated VRAC pore diameter of 1.2–1.4 nm, which is similar to the size of ATP [208], suggesting ATP permeation. Indeed, like the permeating block by calixarenes, VRAC currents induced by hypotonic stress are blocked by extracellular ATP in a voltage-dependent manner in bovine aortic endothelial cells [209]: inhibition of outward but not inward currents through VRACs (i.e., anion influx) progressively strengthens as the membrane voltage increase, reaches a maximum at ~40 mV, and weakens at higher positive potentials. This phenomenon can be explained by the permeating blocker model, where ATP can act as a blocker and permeant at moderate and more depolarized membrane voltages, respectively. Recently, although direct electrophysiological measurements of ATP currents through VRACs are lacking, the ATP permeability of VRACs was assessed by luciferine/luciferase-based measurements of ATP release from *Xenopus* oocytes injected with cRNAs of LRRC8 subunits [198]. Hypotonic exposure promoted ATP release from oocytes expressing untagged LRRC8A/8E in an osmolarity-dependent manner. Further, by employing carboxyl-terminal tagging that enhances the activity of reconstituted VRAC currents, hypotonic stress-induced ATP release was detected from oocytes expressing LRRC8A-VFP/8E-mCherry and 8A-VFP/8C-mCherry but not from oocytes expressing LRRC8A-VFP/8B-mCherry and 8A-VFP/8D-mCherry. Although it is unclear whether the differences in ATP release among LRRC8 heteromers are due to variations in their ATP permeability or simply channel density in the plasma membrane, these observations provide strong evidence for the ATP permeability of VRACs. Although VRACs were originally proposed as a conduit for swelling-induced ATP release [209], accumulating pharmacological evidence [210,211,212,213,214] does not support their involvement. Thus, despite their ATP-releasing function and ubiquitous expression, the roles of ATP released through VRACs remain to be identified.

## 6. Maxi-Anion Channels (MACs)

Since their first description in cultured rat muscle cells in 1983 [215], voltage-dependent, large-conductance, ATP-permeable anion channels, also known as MACs, have been detected in essentially every cell type. However, until the recent discovery of SLCO2A1 as the core subunit of MACs [29], the molecular identity of MACs had remained unknown [216], and they have been functionally defined by a large anion-selective single-channel conductance of 300–500 pS with the permeability sequence of I^−^ > Br^−^ > Cl^−^ > F^−^, a linear current-voltage relationship, voltage-dependent inactivation at positive and negative membrane potentials (i.e., the maximum open probability occurs at ~0 mV), and pharmacological sensitivities distinct from those of other anion channels [217]. MACs are unique among anion channels in their sensitivity to Gd^3+^ [210,218]. MACs are basally silent in resting cells but exhibit multimodal activation due to the presence of various stimuli, including osmotic swelling [210,218,219], salt stress [220], high glucose [221], ischemia [218,222], hypoxia [218], GPCR activation [223,224,225], and excision of a patch membrane [217]. Although it is unclear how MACs are activated by those stimuli, tyrosine phosphorylation [226] and intracellular Ca^2+^ [223] have been suggested to be involved in the regulatory mechanisms.

Recently, Okada and his team [29] discovered SLCO2A1 as the core, pore-forming component of MACs. Among 15 genes that encode proteins with multiple transmembrane-spanning domains yielded by a nano-LC-MS/MS analysis on total bleb-membrane proteins isolated from mouse mammary C127 cells that endogenously express a high level of functional MACs, they identified *Slco2a1* as the gene responsible for MAC activity by siRNA screening. The siRNA- or miRNA-mediated KD targeting of three different sites of *Slco2a1* caused a significant reduction in MAC currents of C127 cells, which could be rescued by the overexpression of a miRNA-insensitive variant of *Slco2a1*, and KO of the gene eliminated the currents. Furthermore, in HEK293T cells lacking SLCO2A1 expression and MAC activity, the exogenous expression of SLCO2A1 generated MAC currents similar to those observed in C127 cells. Importantly, a charge-neutralizing mutation, K613G, reduced the single-channel conductance from 386 to 205 pS and made the channel more selective to cations, suggesting that the residue K613 plays roles in determining the pore properties of the channel. Together with the fact that SLCO2A1 proteins exhibited the activity of MACs when reconstituted into giant proteoliposomes, it was strongly suggested that SLCO2A1 constitutes the pore-forming component of MACs. However, it remains unknown how many SLCO2A1 subunits are contained in a MAC and, thus, how the pore is constructed. Other unknown regulatory components have also been suggested to be involved because MACs formed solely by SLCO2A1 proteins were constitutively active, unlike the basally silent endogenous MACs. Since SLCO2A1 is also known as a prostaglandin transporter, it can switch functions between a channel and transporter.

### 6.1. ATP Permeation through MACs

The permeability of MACs to ATP molecules has been comprehensively established. The first evidence was derived from observations in C127 cells of voltage-dependent open-channel blockade of MACs by ATP applied from either outside or inside, suggesting that ATP interacts with a site located deep inside the pore lumen [210]. In the same study, small inward currents were detected even after the complete replacement of bath Cl^−^ with ATP^4−^ in inside-out patch-clamp recordings. As ATP^4−^ was the only anion in the bath, the recorded unitary inward currents, which were anion-selective and exhibited pharmacological sensitivities identical to those of MACs, had to be carried by ATP^4−^ efflux through a MAC pore [210], establishing MACs as ATP-permeable channels. The permeability ratio of ATP^4−^ to Cl^−^ was calculated as 0.08~0.1. Such direct ATP currents have been recorded in MACs of other cell types [218,220]. Moreover, essentially identical pharmacological profiles between MAC currents and swelling-induced ATP release in C127 cells strongly suggested that MACs are responsible for the swelling-induced ATP release from those cells [210]. Later, pore entrance size estimation (2.3~2.8 nm in diameter) was conducted by the nonelectrolyte exclusion experiments to further support the conclusion that MACs are permeable to ATP because the pore entrance must be wide enough to accommodate ATP molecules [208]. Also, the measurements of permeability to anions with different sizes yielded a pore diameter estimate of >1.1~1.5 nm [227]. Compared with calculated diameters of ATP^4−^ (1.14~1.16 nm) and MgATP^2−^ (1.18~1.22 nm), MACs possess a pore suitable for ATP conduction. The hypotonic cell swelling-induced ATP release was significantly reduced by RNA interference of *Slco2a1* in C127 cells and potentiated by the heterologous expression of SLCO2A1 in HEK293T cells [29], suggesting that MACs constituted by SLCO2A1 are permeable to ATP, although ATP currents through reconstituted SLCO2A1 channels have yet to be demonstrated.

### 6.2. Physiological Roles of MAC-Mediated ATP Release

In addition to its roles in cell-volume regulation and fluid secretion via the transport of Cl^−^ and other small anions, the MAC has also been proposed as a pathway for physiologically important cellular ATP release in specific tissues [217]. In the kidney, changes in the luminal NaCl concentration modify ATP release from the basolateral membranes of macula densa cells located within the thick ascending limb. The released ATP acts on adjacent mesangial cells and possibly on afferent arteriolar smooth muscle cells, and serves as a mediator of tubuloglomerular feedback, which regulates the vascular tone of the afferent arteriole depending on the distal tubule NaCl concentration. ATP-permeable MAC currents were recorded in the basolateral membranes of rabbit macula densa cells and found to be activated by increases in the luminal NaCl concentration, which actually induced basolateral ATP release [220]. Thus, MACs have been suggested to be essential for tubuloglomerular feedback as an ATP-release channel. However, studies using *Slco2a1* KO animals are necessary to support the conclusion. It also remains elusive how macula densa cells sense and transduce changes in the luminal NaCl concentration to MAC activity. Swelling of macula densa cells in response to an increase in the luminal NaCl concentration [228] might be involved. In the ischemic-reperfused heart, the ATP released following reperfusion is known to be cardioprotective [229]. siRNA-mediated KD of *Slco2a1* expression significantly suppressed cardiac ATP release to the coronary effluent following oxygen-glucose deprivation/reperfusion in the Langendorff-perfused adult mouse heart [29], suggesting that MACs play a cardioprotective role as ATP-release channels. In astrocytes, ischemic [222] and hypotonic [230] stresses activate MACs to release ATP, a gliotransmitter linking glial and neuronal cells. The discovery of SLCO2A1 may facilitate the identification of new roles of MACs as physiologically important ATP-release pathways at other sites.

## 7. Concluding Remarks

The five groups of ATP-release channels described above mediate the non-exocytotic, conductive cellular release of ATP in a variety of physiological and pathophysiological systems. As evidenced by CALHM1 [27], ion channels can even mediate action potential-dependent fast purinergic neurotransmission, which had previously been thought to be mediated by exocytotic release. Multiple types of channels often jointly contribute to ATP release in many systems. Because no specific inhibitors have been identified for any of them as yet, pharmacological characterization using non-selective inhibitors and other forms of indirect evidence have comprised the main strategy to assess the contribution of each channel. However, now that genes encoding core subunits of all known ATP-release channels have been cloned, the roles of each channel will be more clearly elucidated through genetic manipulations. Thus, previous pharmacological findings should be re-evaluated to obtain more conclusive evidence. However, the development of specific inhibitors is still an urgent task for drug development, as ATP-release channels have been reported to be associated with various diseases. The regulatory mechanisms of these channels, especially those that have recently been cloned, remain largely elusive. There are still unknown mechanisms of physiologically important ATP release. Armed with molecular information, we now can attempt to clarify these mechanisms more strategically than ever.

## Abbreviations

ABC	ATP-binding cassette transporter
ATP	adenosine triphosphate
CALHM1	calcium homeostasis modulator 1
CFTR	cystic fibrosis transmembrane conductance regulator
[Ca^2+^]_o_	extracellular Ca^2+^ concentration
K^+^_o_	extracellular K^+^
Ca^2+^_i_	intracellular Ca^2+^
KD	knockdown
KO	knockout
MAC	maxi-anion channel
*P_o_*	open probability
PANX1	pannexin 1
VRAC	volume-regulated anion channel
VSOR	volume-sensitive outwardly rectifying
